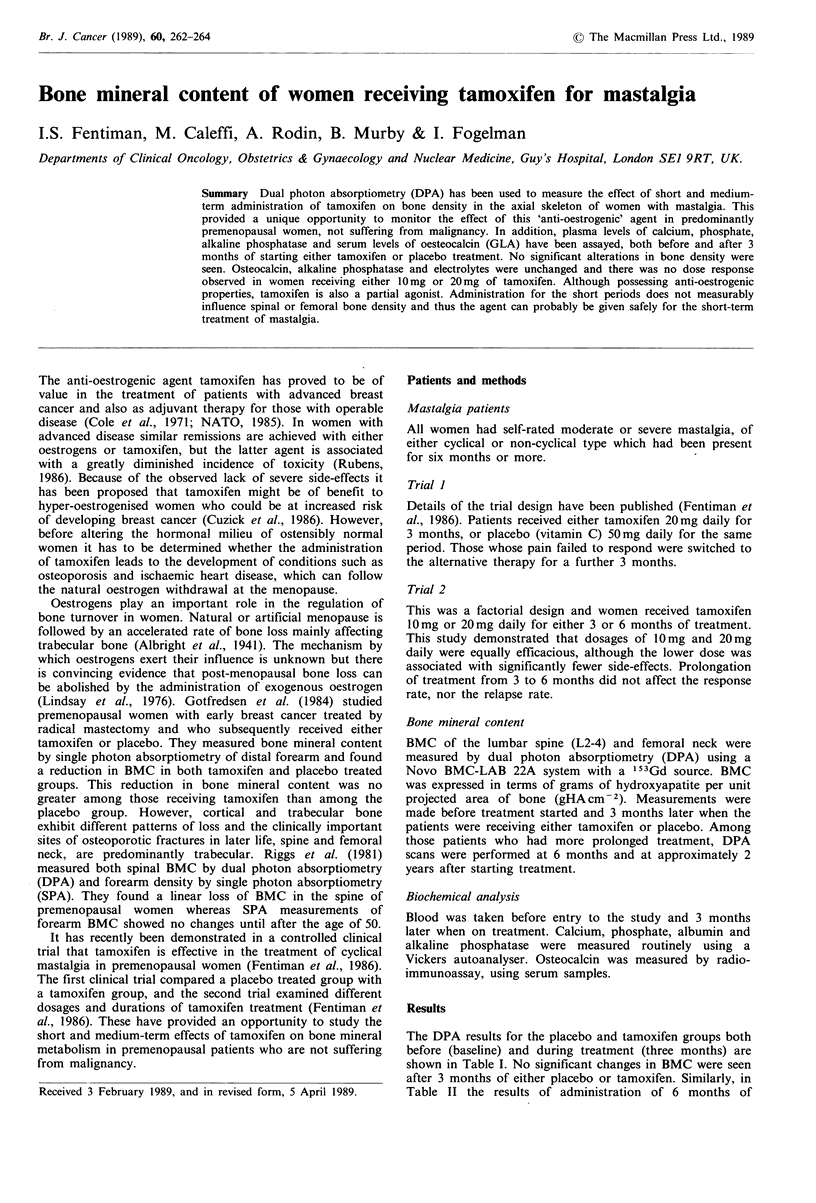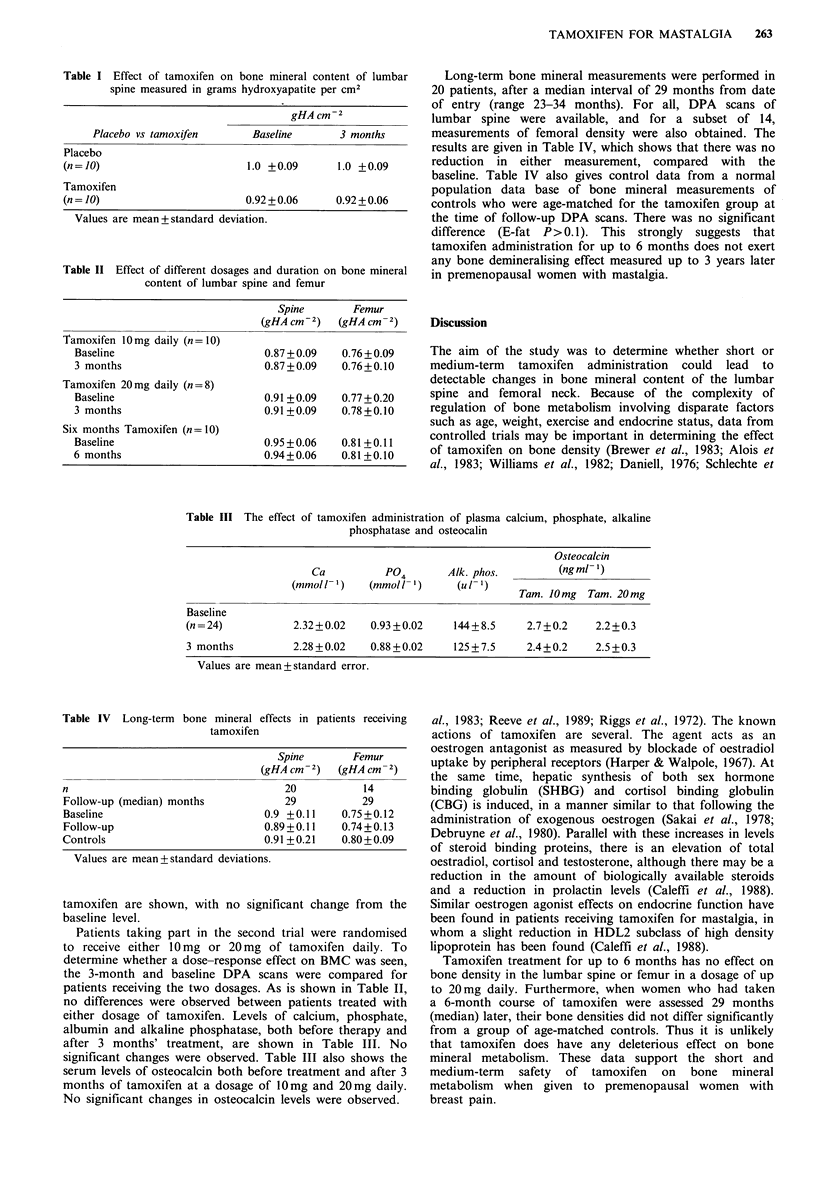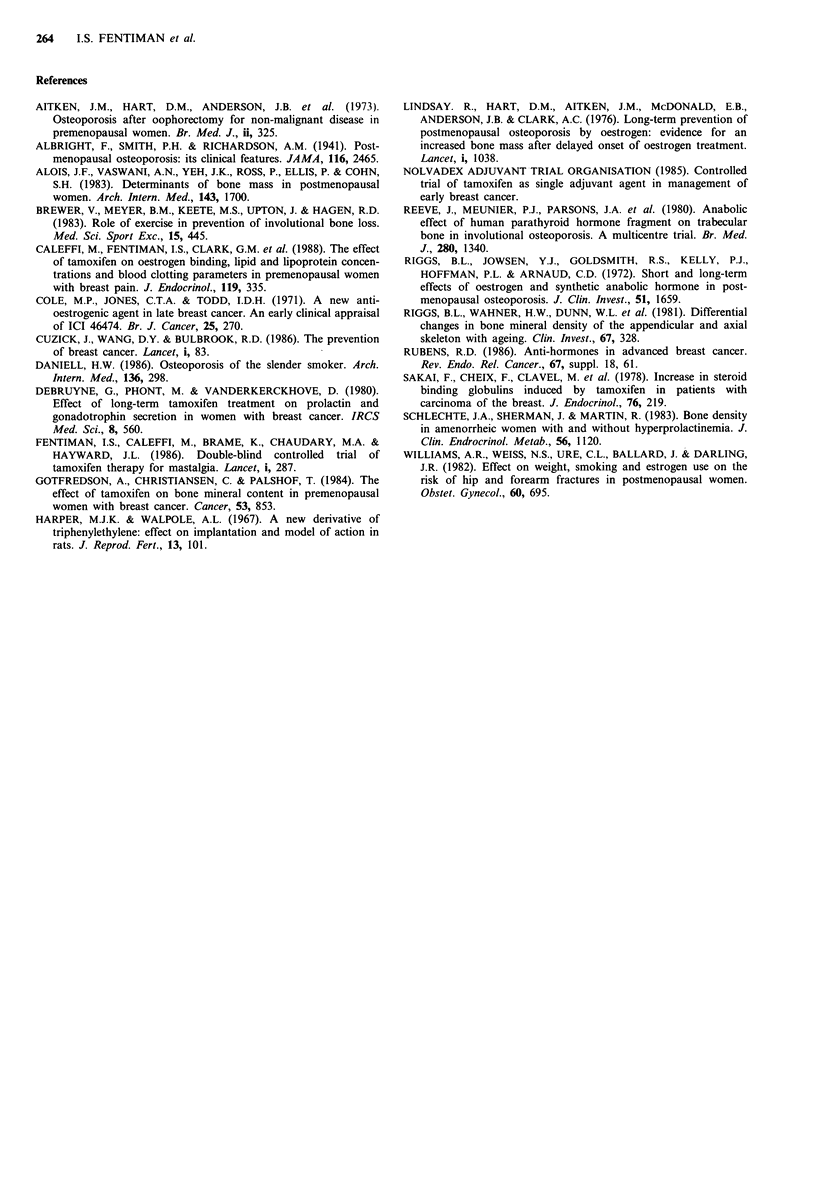# Bone mineral content of women receiving tamoxifen for mastalgia.

**DOI:** 10.1038/bjc.1989.266

**Published:** 1989-08

**Authors:** I. S. Fentiman, M. Caleffi, A. Rodin, B. Murby, I. Fogelman

**Affiliations:** Department of Clinical Oncology, Guy's Hospital, London, UK.

## Abstract

Dual photon absorptiometry (DPA) has been used to measure the effect of short and medium-term administration of tamoxifen on bone density in the axial skeleton of women with mastalgia. This provided a unique opportunity to monitor the effect of this 'anti-oestrogenic' agent in predominantly premenopausal women, not suffering from malignancy. In addition, plasma levels of calcium, phosphate, alkaline phosphatase and serum levels of oesteocalcin (GLA) have been assayed, both before and after 3 months of starting either tamoxifen or placebo treatment. No significant alterations in bone density were seen. Osteocalcin, alkaline phosphatase and electrolytes were unchanged and there was no dose response observed in women receiving either 10 mg or 20 mg of tamoxifen. Although possessing anti-oestrogenic properties, tamoxifen is also a partial agonist. Administration for the short periods does not measurably influence spinal or femoral bone density and thus the agent can probably be given safely for the short-term treatment of mastalgia.


Br. J. Cancer (1989), 60, 262-264 © The Macmillan Press Ltd., 1989

### Bone mineral content of women receiving tamoxifen for mastalgia

I.S. Fentiman, M. Caleffi, A. Rodin, B. Murby & I. Fogelman

*Departments of Clinical Oncology, Obstetrics & Gynaecology and Nuclear Medicine, Guy's Hospital, London SE1 9RT, UK.*

**Summary** Dual photon absorptiometry (DPA) has been used to measure the effect of short and medium-term administration of tamoxifen on bone density in the axial skeleton of women with mastalgia. This provided a unique opportunity to monitor the effect of this 'anti-oestrogenic' agent in predominantly premenopausal women, not suffering from malignancy. In addition, plasma levels of calcium, phosphate, alkaline phosphatase and serum levels of oesteocalcin (GLA) have been assayed, both before and after 3 months of starting either tamoxifen or placebo treatment. No significant alterations in bone density were seen. Osteocalcin, alkaline phosphatase and electrolytes were unchanged and there was no dose response observed in women receiving either 10 mg or 20 mg of tamoxifen. Although possessing anti-oestrogenic properties, tamoxifen is also a partial agonist. Administration for the short periods does not measurably influence spinal or femoral bone density and thus the agent can probably be given safely for the short-term treatment of mastalgia.

The anti-oestrogenic agent tamoxifen has proved to be of value in the treatment of patients with advanced breast cancer and also as adjuvant therapy for those with operable disease (Cole *et al.*, 1971; NATO, 1985). In women with advanced disease similar remissions are achieved with either oestrogens or tamoxifen, but the latter agent is associated with a greatly diminished incidence of toxicity (Rubens, 1986). Because of the observed lack of severe side-effects it has been proposed that tamoxifen might be of benefit to hyper-oestrogenised women who could be at increased risk of developing breast cancer (Cuzick *et al.*, 1986). However, before altering the hormonal milieu of ostensibly normal women it has to be determined whether the administration of tamoxifen leads to the development of conditions such as osteoporosis and ischaemic heart disease, which can follow the natural oestrogen withdrawal at the menopause.

Oestrogens play an important role in the regulation of bone turnover in women. Natural or artificial menopause is followed by an accelerated rate of bone loss mainly affecting trabecular bone (Albright *et al.*, 1941). The mechanism by which oestrogens exert their influence is unknown but there is convincing evidence that post-menopausal bone loss can be abolished by the administration of exogenous oestrogen (Lindsay *et al.*, 1976). Gotfredsen *et al.* (1984) studied premenopausal women with early breast cancer treated by radical mastectomy and who subsequently received either tamoxifen or placebo. They measured bone mineral content by single photon absorptiometry of distal forearm and found a reduction in BMC in both tamoxifen and placebo treated groups. This reduction in bone mineral content was no greater among those receiving tamoxifen than among the placebo group. However, cortical and trabecular bone exhibit different patterns of loss and the clinically important sites of osteoporotic fractures in later life, spine and femoral neck, are predominantly trabecular. Riggs *et al.* (1981) measured both spinal BMC by dual photon absorptiometry (DPA) and forearm density by single photon absorptiometry (SPA). They found a linear loss of BMC in the spine of premenopausal women whereas SPA measurements of forearm BMC showed no changes until after the age of 50.

It has recently been demonstrated in a controlled clinical trial that tamoxifen is effective in the treatment of cyclical mastalgia in premenopausal women (Fentiman *et al.*, 1986). The first clinical trial compared a placebo treated group with a tamoxifen group, and the second trial examined different dosages and durations of tamoxifen treatment (Fentiman *et al.*, 1986). These have provided an opportunity to study the short and medium-term effects of tamoxifen on bone mineral metabolism in premenopausal patients who are not suffering from malignancy.

Received 3 February 1989, and in revised form, 5 April 1989.

### Patients and methods

### *Mastalgia patients*

All women had self-rated moderate or severe mastalgia, of either cyclical or non-cyclical type which had been present for six months or more.

### *Trial 1*

Details of the trial design have been published (Fentiman *et al.*, 1986). Patients received either tamoxifen 20 mg daily for 3 months, or placebo (vitamin C) 50 mg daily for the same period. Those whose pain failed to respond were switched to the alternative therapy for a further 3 months.

### *Trial 2*

This was a factorial design and women received tamoxifen 10 mg or 20 mg daily for either 3 or 6 months of treatment. This study demonstrated that dosages of 10 mg and 20 mg daily were equally efficacious, although the lower dose was associated with significantly fewer side-effects. Prolongation of treatment from 3 to 6 months did not affect the response rate, nor the relapse rate.

### *Bone mineral content*

BMC of the lumbar spine (L2-4) and femoral neck were measured by dual photon absorptiometry (DPA) using a Novo BMC-LAB 22A system with a $^{153}$Gd source. BMC was expressed in terms of grams of hydroxyapatite per unit projected area of bone (gHA $cm^{-2}$). Measurements were made before treatment started and 3 months later when the patients were receiving either tamoxifen or placebo. Among those patients who had more prolonged treatment, DPA scans were performed at 6 months and at approximately 2 years after starting treatment.

### *Biochemical analysis*

Blood was taken before entry to the study and 3 months later when on treatment. Calcium, phosphate, albumin and alkaline phosphatase were measured routinely using a Vickers autoanalyser. Osteocalcin was measured by radioimmunoassay, using serum samples.

### Results

The DPA results for the placebo and tamoxifen groups both before (baseline) and during treatment (three months) are shown in Table I. No significant changes in BMC were seen after 3 months of either placebo or tamoxifen. Similarly, in Table II the results of administration of 6 months of

**Table I** Effect of tamoxifen on bone mineral content of lumbar spine measured in grams hydroxyapatite per cm²

| | $gHA\,cm^{-2}$ | |
|---|---|---|
| *Placebo vs tamoxifen* | *Baseline* | *3 months* |
| Placebo ($n=10$) | 1.0 ±0.09 | 1.0 ±0.09 |
| Tamoxifen ($n=10$) | 0.92±0.06 | 0.92±0.06 |

Values are mean±standard deviation.

**Table II** Effect of different dosages and duration on bone mineral content of lumbar spine and femur

| | *Spine* ($gHA\,cm^{-2}$) | *Femur* ($gHA\,cm^{-2}$) |
|---|---|---|
| Tamoxifen 10mg daily ($n=10$) | | |
| Baseline | 0.87±0.09 | 0.76±0.09 |
| 3 months | 0.87±0.09 | 0.76±0.10 |
| Tamoxifen 20mg daily ($n=8$) | | |
| Baseline | 0.91±0.09 | 0.77±0.20 |
| 3 months | 0.91±0.09 | 0.78±0.10 |
| Six months Tamoxifen ($n=10$) | | |
| Baseline | 0.95±0.06 | 0.81±0.11 |
| 6 months | 0.94±0.06 | 0.81±0.10 |

**Table III** The effect of tamoxifen administration of plasma calcium, phosphate, alkaline phosphatase and osteocalin

| | *Ca* ($mmol\,l^{-1}$) | $PO_4$ ($mmol\,l^{-1}$) | *Alk. phos.* ($u\,l^{-1}$) | *Osteocalcin* ($ng\,ml^{-1}$) | |
|---|---|---|---|---|---|
| | | | | *Tam. 10mg* | *Tam. 20mg* |
| Baseline ($n=24$) | 2.32±0.02 | 0.93±0.02 | 144±8.5 | 2.7±0.2 | 2.2±0.3 |
| 3 months | 2.28±0.02 | 0.88±0.02 | 125±7.5 | 2.4±0.2 | 2.5±0.3 |

Values are mean±standard error.

**Table IV** Long-term bone mineral effects in patients receiving tamoxifen

| | *Spine* ($gHA\,cm^{-2}$) | *Femur* ($gHA\,cm^{-2}$) |
|---|---|---|
| *n* | 20 | 14 |
| Follow-up (median) months | 29 | 29 |
| Baseline | 0.9 ±0.11 | 0.75±0.12 |
| Follow-up | 0.89±0.11 | 0.74±0.13 |
| Controls | 0.91±0.21 | 0.80±0.09 |

Values are mean±standard deviations.

tamoxifen are shown, with no significant change from the baseline level.

Patients taking part in the second trial were randomised to receive either 10mg or 20mg of tamoxifen daily. To determine whether a dose–response effect on BMC was seen, the 3-month and baseline DPA scans were compared for patients receiving the two dosages. As is shown in Table II, no differences were observed between patients treated with either dosage of tamoxifen. Levels of calcium, phosphate, albumin and alkaline phosphatase, both before therapy and after 3 months' treatment, are shown in Table III. No significant changes were observed. Table III also shows the serum levels of osteocalcin both before treatment and after 3 months of tamoxifen at a dosage of 10mg and 20mg daily. No significant changes in osteocalcin levels were observed.

Long-term bone mineral measurements were performed in 20 patients, after a median interval of 29 months from date of entry (range 23–34 months). For all, DPA scans of lumbar spine were available, and for a subset of 14, measurements of femoral density were also obtained. The results are given in Table IV, which shows that there was no reduction in either measurement, compared with the baseline. Table IV also gives control data from a normal population data base of bone mineral measurements of controls who were age-matched for the tamoxifen group at the time of follow-up DPA scans. There was no significant difference (E-fat $P>0.1$). This strongly suggests that tamoxifen administration for up to 6 months does not exert any bone demineralising effect measured up to 3 years later in premenopausal women with mastalgia.

### Discussion

The aim of the study was to determine whether short or medium-term tamoxifen administration could lead to detectable changes in bone mineral content of the lumbar spine and femoral neck. Because of the complexity of regulation of bone metabolism involving disparate factors such as age, weight, exercise and endocrine status, data from controlled trials may be important in determining the effect of tamoxifen on bone density (Brewer *et al.*, 1983; Alois *et al.*, 1983; Williams *et al.*, 1982; Daniell, 1976; Schlechte *et al.*, 1983; Reeve *et al.*, 1989; Riggs *et al.*, 1972). The known actions of tamoxifen are several. The agent acts as an oestrogen antagonist as measured by blockade of oestradiol uptake by peripheral receptors (Harper & Walpole, 1967). At the same time, hepatic synthesis of both sex hormone binding globulin (SHBG) and cortisol binding globulin (CBG) is induced, in a manner similar to that following the administration of exogenous oestrogen (Sakai *et al.*, 1978; Debruyne *et al.*, 1980). Parallel with these increases in levels of steroid binding proteins, there is an elevation of total oestradiol, cortisol and testosterone, although there may be a reduction in the amount of biologically available steroids and a reduction in prolactin levels (Caleffi *et al.*, 1988). Similar oestrogen agonist effects on endocrine function have been found in patients receiving tamoxifen for mastalgia, in whom a slight reduction in HDL2 subclass of high density lipoprotein has been found (Caleffi *et al.*, 1988).

Tamoxifen treatment for up to 6 months has no effect on bone density in the lumbar spine or femur in a dosage of up to 20mg daily. Furthermore, when women who had taken a 6-month course of tamoxifen were assessed 29 months (median) later, their bone densities did not differ significantly from a group of age-matched controls. Thus it is unlikely that tamoxifen does have any deleterious effect on bone mineral metabolism. These data support the short and medium-term safety of tamoxifen on bone mineral metabolism when given to premenopausal women with breast pain.

### References

AITKEN, J.M., HART, D.M., ANDERSON, J.B. *et al.* (1973). Osteoporosis after oophorectomy for non-malignant disease in premenopausal women. *Br. Med. J.*, **ii**, 325.

ALBRIGHT, F., SMITH, P.H. & RICHARDSON, A.M. (1941). Post-menopausal osteoporosis: its clinical features. *JAMA*, **116**, 2465.

ALOIS, J.F., VASWANI, A.N., YEH, J.K., ROSS, P., ELLIS, P. & COHN, S.H. (1983). Determinants of bone mass in postmenopausal women. *Arch. Intern. Med.*, **143**, 1700.

BREWER, V., MEYER, B.M., KEETE, M.S., UPTON, J. & HAGEN, R.D. (1983). Role of exercise in prevention of involutional bone loss. *Med. Sci. Sport Exc.*, **15**, 445.

CALEFFI, M., FENTIMAN, I.S., CLARK, G.M. *et al.* (1988). The effect of tamoxifen on oestrogen binding, lipid and lipoprotein concentrations and blood clotting parameters in premenopausal women with breast pain. *J. Endocrinol.*, **119**, 335.

COLE, M.P., JONES, C.T.A. & TODD, I.D.H. (1971). A new anti-oestrogenic agent in late breast cancer. An early clinical appraisal of ICI 46474. *Br. J. Cancer*, **25**, 270.

CUZICK, J., WANG, D.Y. & BULBROOK, R.D. (1986). The prevention of breast cancer. *Lancet*, **i**, 83.

DANIELL, H.W. (1986). Osteoporosis of the slender smoker. *Arch. Intern. Med.*, **136**, 298.

DEBRUYNE, G., PHONT, M. & VANDERKERCKHOVE, D. (1980). Effect of long-term tamoxifen treatment on prolactin and gonadotrophin secretion in women with breast cancer. *IRCS Med. Sci.*, **8**, 560.

FENTIMAN, I.S., CALEFFI, M., BRAME, K., CHAUDARY, M.A. & HAYWARD, J.L. (1986). Double-blind controlled trial of tamoxifen therapy for mastalgia. *Lancet*, **i**, 287.

GOTFREDSON, A., CHRISTIANSEN, C. & PALSHOF, T. (1984). The effect of tamoxifen on bone mineral content in premenopausal women with breast cancer. *Cancer*, **53**, 853.

HARPER, M.J.K. & WALPOLE, A.L. (1967). A new derivative of triphenylethylene: effect on implantation and model of action in rats. *J. Reprod. Fert.*, **13**, 101.

LINDSAY. R., HART, D.M., AITKEN, J.M., McDONALD, E.B., ANDERSON, J.B. & CLARK, A.C. (1976). Long-term prevention of postmenopausal osteoporosis by oestrogen: evidence for an increased bone mass after delayed onset of oestrogen treatment. *Lancet*, **i**, 1038.

NOLVADEX ADJUVANT TRIAL ORGANISATION (1985). Controlled trial of tamoxifen as single adjuvant agent in management of early breast cancer.

REEVE, J., MEUNIER, P.J., PARSONS, J.A. *et al.* (1980). Anabolic effect of human parathyroid hormone fragment on trabecular bone in involutional osteoporosis. A multicentre trial. *Br. Med. J.*, **280**, 1340.

RIGGS, B.L., JOWSEN, Y.J., GOLDSMITH, R.S., KELLY, P.J., HOFFMAN, P.L. & ARNAUD, C.D. (1972). Short and long-term effects of oestrogen and synthetic anabolic hormone in post-menopausal osteoporosis. *J. Clin. Invest.*, **51**, 1659.

RIGGS, B.L., WAHNER, H.W., DUNN, W.L. *et al.* (1981). Differential changes in bone mineral density of the appendicular and axial skeleton with ageing. *Clin. Invest.*, **67**, 328.

RUBENS, R.D. (1986). Anti-hormones in advanced breast cancer. *Rev. Endo. Rel. Cancer.*, **67**, suppl. 18, 61.

SAKAI, F., CHEIX, F., CLAVEL, M. *et al.* (1978). Increase in steroid binding globulins induced by tamoxifen in patients with carcinoma of the breast. *J. Endocrinol.*, **76**, 219.

SCHLECHTE, J.A., SHERMAN, J. & MARTIN, R. (1983). Bone density in amenorrheic women with and without hyperprolactinemia. *J. Clin. Endrocrinol. Metab.*, **56**, 1120.

WILLIAMS, A.R., WEISS, N.S., URE, C.L., BALLARD, J. & DARLING, J.R. (1982). Effect on weight, smoking and estrogen use on the risk of hip and forearm fractures in postmenopausal women. *Obstet. Gynecol.*, **60**, 695.